# Reconstructing Hope Through Horticultural Therapy: A Phenomenological Study of Individuals with Post-Stroke Depression

**DOI:** 10.3390/healthcare14193229

**Published:** 2026-10-01

**Authors:** Yuehua Gong, Haijian Shen, Lingli Ren, Xiaoping Zhu

**Affiliations:** 1 Shanghai Jing’an District Shibei Hospital, Shanghai 200443, China; 2441005@tongji.edu.cn (Y.G.); sbhlgl@163.com (H.S.); 2 Tongji University School of Medicine, Shanghai 200092, China; 3 Shanghai Tenth People’s Hospital, Shanghai 200072, China; 2440982@tongji.edu.cn

**Keywords:** post-stroke depression, horticultural therapy, empowerment, psychological recovery, psychosocial rehabilitation, qualitative research

## Abstract

**Background:** Post-stroke depression (PSD) is a common psychological complication that negatively affects rehabilitation and quality of life. This study explored the lived experiences of patients with PSD participating in horticultural therapy (HT). **Methods:** A descriptive phenomenological study was conducted among 15 individuals with mild-to-moderate PSD who completed a six-month HT program in Shanghai, China. Semi-structured interviews were analyzed using Colaizzi’s seven-step method. **Results:** Five themes emerged: rebuilding self-efficacy, reconstructing hope, enhancing social connectedness and sense of value, facilitating physical and psychological rehabilitation, and challenges during horticultural activities. Participants described HT as a meaningful experience that fostered confidence, hope, social participation, and motivation for recovery. Plant growth was often interpreted by participants as a symbol of personal resilience and hope, while horticultural activities were described as meaningful experiences that fostered social interaction and a sense of purpose. Nevertheless, plant loss and physical limitations sometimes generated frustration and helplessness. **Conclusions:** Participants described HT as a meaningful experience associated with self-efficacy, hope, social connectedness, and engagement in rehabilitation. The findings highlight the potential value of individualized horticultural activities as a supportive component of care for individuals with PSD and may inform the development of person-centered rehabilitation programs.

## 1. Introduction

Post-stroke depression (PSD) is one of the most common neuropsychiatric complications following stroke, affecting approximately one-third of stroke survivors during the recovery process [1,2]. PSD is characterized by persistent low mood, loss of interest, reduced motivation, and emotional distress, all of which may substantially hinder neurological recovery, functional rehabilitation, and social reintegration [1,2,3]. Previous studies have demonstrated that PSD is associated with poorer functional outcomes, lower quality of life, increased disability, prolonged hospitalization, and higher mortality among stroke survivors [1,2,3,4]. Beyond its impact on physical recovery, PSD may also reduce patients’ willingness to participate in rehabilitation activities, thereby creating a vicious cycle that further compromises long-term recovery outcomes [3,4,5,6].

Pharmacological treatment, particularly antidepressant therapy, remains the primary intervention for PSD. Although antidepressants have demonstrated effectiveness in reducing depressive symptoms, their benefits are often limited by delayed onset of action, adverse effects, medication burden, and poor treatment adherence [6,7]. Furthermore, stroke survivors frequently experience multiple comorbidities and are often prescribed several medications simultaneously, which may further increase concerns related to polypharmacy and long-term medication use [6]. Consequently, there is growing interest in identifying safe, accessible, and person-centered non-pharmacological interventions that can complement conventional treatment and support psychological recovery after stroke [7,8].

Horticultural therapy (HT) is a nature-based therapeutic intervention that utilizes horticultural activities, plant cultivation, and interactions with natural environments to promote physical, psychological, and social well-being [9]. In recent years, HT has gained increasing attention in rehabilitation and mental healthcare settings because of its potential to provide meaningful engagement, sensory stimulation, and opportunities for achievement and social participation [10]. Evidence from systematic reviews and meta-analyses suggests that horticultural activities may reduce symptoms of depression and anxiety, improve emotional well-being, enhance social connectedness, and increase overall life satisfaction across diverse populations [9,10,11]. Among individuals undergoing rehabilitation, previous studies have reported positive experiences related to motivation, engagement in therapeutic activities, and quality of life among participants involved in horticultural therapy programs [10,12].

Several theoretical perspectives may explain the beneficial effects of horticultural therapy. Attention Restoration Theory proposes that exposure to natural environments can restore depleted cognitive resources and alleviate mental fatigue, thereby promoting psychological recovery [13]. Similarly, the Biophilia Hypothesis suggests that human beings possess an innate tendency to seek connections with nature and other living organisms, resulting in positive psychological responses during interactions with natural environments [14,15]. Beyond environmental influences, horticultural activities also provide opportunities for mastery experiences, goal achievement, and social interaction, all of which may contribute to enhanced self-efficacy and psychological well-being [10].

Despite the growing evidence supporting the effectiveness of horticultural therapy, existing research has primarily focused on quantitative outcomes, such as changes in depression scores, psychological well-being, cognitive function, and quality of life [9,10,11,12]. Although such findings demonstrate the potential benefits of HT, they provide limited insight into how participants experience the intervention and how these experiences influence recovery processes. In particular, little is known about the subjective experiences of patients with PSD during participation in horticultural therapy. Understanding how patients interpret plant growth, perceive the therapeutic process, and construct meaning from horticultural activities may provide valuable information regarding the mechanisms underlying therapeutic effectiveness.

Empowerment Theory offers a useful framework for exploring these experiences. Empowerment refers to a process through which individuals gain greater control over decisions and actions affecting their health and well-being. The theory emphasizes both internal empowerment, including self-efficacy, confidence, hope, and self-awareness, and external empowerment, including social support, access to resources, and meaningful participation within supportive environments [16]. For individuals with PSD, whose experiences are often characterized by loss of independence, reduced self-worth, and social withdrawal, empowerment may represent a critical pathway toward psychological adaptation and rehabilitation engagement. Horticultural therapy has the potential to facilitate both internal and external empowerment by providing opportunities for caregiving, achievement, social interaction, and personal growth.

Therefore, guided by Empowerment Theory, this descriptive phenomenological study aimed to explore the lived experiences of patients with post-stroke depression participating in horticultural therapy. In this study, Empowerment Theory informed interview development and guided interpretation, while themes were inductively derived from participants’ narratives. By examining participants’ perceptions, emotional responses, and rehabilitation experiences, this study sought to deepen understanding of the mechanisms through which horticultural therapy supports psychological recovery and to provide evidence for optimizing nature-based interventions within comprehensive stroke rehabilitation programs.

## 2. Materials and Methods

### 2.1. Study Design

This study employed a descriptive phenomenological approach to explore the lived experiences of patients with PSD who participated in a horticultural therapy program. Descriptive phenomenology seeks to understand and describe the essential meaning of individuals’ experiences of a particular phenomenon as perceived from their own perspectives. This methodological approach was considered appropriate because the aim of the study was to gain an in-depth understanding of how patients experienced horticultural therapy and how they perceived its influence on their psychological recovery and rehabilitation process.

Empowerment Theory served as the theoretical framework for this study and informed the development of the interview guide. However, data collection and analysis were conducted using a descriptive phenomenological approach and Colaizzi’s seven-step method. Themes were derived inductively from participants’ narratives rather than being predetermined by Empowerment Theory. The theory was primarily used to support the interpretation and discussion of the findings. In this study, Empowerment Theory informed interview development and guided interpretation, while themes were inductively derived from participants’ narratives. The study was reported in accordance with the Consolidated Criteria for Reporting Qualitative Research (COREQ) [17].

### 2.2. Participants and Recruitment

Participants were recruited using purposive sampling from the Department of Neurology of Shibei Hospital, a secondary general hospital in Shanghai, China, between June and October 2025. Semi-structured interviews were conducted within two weeks following completion of the horticultural therapy program.

The inclusion criteria were as follows:

1. A diagnosis of post-stroke depression (PSD) according to the Chinese Guideline for the Prevention and Treatment of Post-Stroke Depression [18], confirmed by experienced rehabilitation physicians based on clinical assessment and medical records, and a 17-item Hamilton Depression Rating Scale (HAMD-17) score between 8 and 24, indicating mild to moderate depressive symptoms and ensuring that participants were able to engage meaningfully in interviews and horticultural therapy activities [19]. HAMD-17 assessments were conducted by trained rehabilitation clinicians prior to enrollment.
2. First episode of PSD following stroke.
3. Stable medical condition with clear consciousness and the ability to communicate effectively.
4. Completion of at least six months of participation in the horticultural therapy programme.
5. Willingness to participate and provide written informed consent.

The exclusion criteria were:

1. Severe cognitive impairment, aphasia, or communication difficulties that could affect participation in interviews.
2. Presence of severe psychiatric disorders other than depression.
3. Activities of Daily Living (ADL) score ≤ 40 or muscle strength ≤ Grade III.
4. Serious medical conditions that could significantly affect participation in rehabilitation activities.

All participants had been diagnosed with PSD after stroke onset and prior to enrollment in the study. During the study period, participants continued to receive standard clinical care, including routine rehabilitation services and any pharmacological or psychological treatments prescribed by their treating clinicians. Horticultural therapy was delivered as a complementary intervention and did not replace usual medical or rehabilitation care. Review of available medical records indicated that no major changes in depression-related treatment occurred during the six-month horticultural therapy period.

The horticultural therapy programme was not part of the hospital’s existing rehabilitation services and was implemented specifically for research purposes. Participants engaged in the programme as part of their rehabilitation care. The present study aimed to explore patients’ lived experiences of participating in the programme.

Information on concurrent antidepressant medication or psychotherapy was not systematically collected as part of this qualitative study. A review of available medical records indicated that no major changes in depression-related treatment occurred during the six-month horticultural therapy period.

Participant recruitment continued until data saturation was achieved. Data collection and preliminary analysis were conducted concurrently. Data saturation was defined as the point at which no new themes, categories, or meaningful insights emerged from subsequent interviews. After the thirteenth interview, no substantially new meanings or themes emerged. Two additional interviews were conducted to confirm thematic completeness. Data saturation was therefore considered achieved after the fifteenth interview, and a total of 15 participants were included in the final analysis.

Participants who had completed at least six months of participation in the horticultural therapy programme were eligible for recruitment. Purposive sampling was used to identify participants who met the study eligibility criteria and were willing to share their experiences of participating in the horticultural therapy programme. Eligible participants were recruited between June and October 2025. Data collection was conducted during the same period.

### 2.3. Horticultural Therapy Intervention

Participants engaged in a structured horticultural therapy program lasting six months. Sessions were conducted once weekly and lasted approximately 60–90 min. The program was developed and delivered by a multidisciplinary team consisting of nurses, rehabilitation therapists, horticultural therapy instructors, and psychological counselors. All facilitators received training regarding the intervention procedures and participant safety prior to program implementation.

The intervention included a range of horticultural activities such as seed sowing, transplanting, watering, pruning, fertilizing, flower arrangement, plant maintenance, and plant-related handicraft production. Activities were progressively adjusted according to participants’ physical abilities and rehabilitation goals.

The core components of the intervention were delivered consistently across participants; however, specific activities and task difficulty were adapted according to individual functional abilities, rehabilitation needs, and personal preferences. Sessions were conducted in small groups to promote social interaction and peer support. Activities took place in designated horticultural spaces within the rehabilitation setting, and seasonal factors were considered when selecting planting and cultivation tasks.

Participants were encouraged to take responsibility for caring for their plants throughout the intervention period. Healthcare professionals provided guidance and support while promoting autonomy and active participation. Group discussions, product exhibitions, and sharing activities were incorporated to facilitate social interaction, emotional expression, and peer support. Attendance and participation were monitored throughout the intervention period.

Attendance was recorded throughout the intervention, and missed sessions were documented by the intervention team. Participants were also encouraged, when feasible, to continue plant-care activities outside scheduled sessions to enhance engagement with the program.

Unlike general recreational horticultural activities, the horticultural therapy program was a structured therapeutic intervention designed to support psychological well-being, social participation, and engagement in rehabilitation through meaningful interaction with living plants.

### 2.4. Data Collection

Data were collected through semi-structured, face-to-face interviews. An interview guide was developed based on Empowerment Theory, findings from previous studies, and consultation with experts in nursing, psychology, and rehabilitation medicine.

Although Empowerment Theory informed the development of the interview guide, the interviews were intended to explore participants’ lived experiences rather than to confirm predefined benefits of horticultural therapy. Open-ended questions and flexible probing were used to encourage participants to discuss positive, negative, or neutral aspects of their experiences. Themes were derived inductively from participants’ narratives rather than being predetermined by the theoretical framework.

Participants were encouraged to share any experiences they considered meaningful during the horticultural therapy program, regardless of whether those experiences were perceived as beneficial or challenging.

The main interview questions included:

1. How have your emotions and psychological state changed since participating in horticultural activities?
2. Can you describe any memorable experiences during the process of planting or caring for plants?
3. In what ways, if any, do you believe horticultural therapy has influenced your rehabilitation?
4. Have you encountered any difficulties or frustrations during the horticultural activities? How did you deal with them?
5. How would you describe the relationship between yourself and the plants you cared for?
6. What aspects of the horticultural activities were most meaningful to you?

All interviews were conducted by researchers trained in qualitative interviewing techniques. Interviews took place in a private and quiet room within the hospital to minimize interruptions and ensure participant comfort.

Prior to each interview, participants were informed about the study objectives and interview procedures. Written informed consent was obtained before data collection. Interviews lasted approximately 30–50 min and were audio-recorded with participants’ permission.

Field notes were recorded immediately after each interview to document contextual information, non-verbal behaviors, emotional reactions, and researchers’ preliminary reflections.

Data collection and analysis were conducted simultaneously, allowing emerging themes to inform subsequent interviews. The core interview guide remained unchanged throughout the study. However, additional probing questions were used when appropriate to explore emerging topics raised by participants in greater depth.

The interviewer (H.S.) was a rehabilitation therapist with experience in stroke rehabilitation and qualitative interviewing. Prior to data collection, no therapeutic or professional relationship existed between the interviewer and participants. The interviewer was not involved in the design, delivery, supervision, or evaluation of the horticultural therapy programme.

The research team included researchers with expertise in nursing, rehabilitation medicine, and qualitative research. Data analysis was conducted independently by Y.G., a master’s student, and H.S., both of whom had received qualitative research training and had experience in qualitative health research. Data interpretation and theme refinement were supported by L.R., a registered nurse, and X.Z., a professor of nursing with extensive experience in qualitative research and nursing education. Regular research meetings were conducted throughout the study to discuss coding decisions, resolve discrepancies, and enhance analytical rigor.

Throughout the study, reflexive journals were maintained as part of a bracketing process to document assumptions, methodological decisions, and emerging interpretations. Regular discussions were conducted within the research team to enhance reflexivity and minimize potential researcher bias.

The complete semi-structured interview guide is provided in Supplementary Material S1.

### 2.5. Data Analysis

Audio recordings were transcribed verbatim within 24 h following each interview. The transcripts were repeatedly reviewed against the original recordings to ensure accuracy.

Data were analyzed according to Colaizzi’s seven-step descriptive phenomenological method [20]:

1. Reading all transcripts repeatedly to gain an overall understanding of participants’ experiences;
2. Extracting significant statements relevant to the phenomenon under investigation;
3. Formulating meanings from significant statements;
4. Organizing formulated meanings into clusters of themes;
5. Developing an exhaustive description of the phenomenon;
6. Identifying the fundamental structure of the experience;
7. Returning findings to participants for member checking and validation.

Two researchers independently analyzed the interview data using Colaizzi’s seven-step phenomenological method. Differences in interpretation were discussed within the research team until consensus was reached. An audit trail was maintained throughout the analytical process. Significant statements were identified from the transcripts and transformed into formulated meanings. Similar meanings were subsequently grouped into preliminary codes, which were organized into subthemes and broader themes through iterative comparison and discussion. Throughout the analytical process, regular discussions among the research team were undertaken to review interpretations, refine theme clusters, and ensure consistency and rigor in the application of Colaizzi’s phenomenological method. The development of themes was documented at each stage to ensure transparency and traceability between the original data and the final thematic structure. An example illustrating the development of significant statements into formulated meanings, codes, subthemes, and themes is provided in Supplementary Material S2.

Member checking was conducted with participants who agreed to take part in the validation process. Participants were provided with a summary of the preliminary themes and interpretations and were invited to comment on the accuracy, completeness, and relevance of the findings. Participants generally confirmed that the findings reflected their experiences. Minor clarifications regarding wording and expression were suggested; however, no substantial changes to the thematic structure or interpretation of the findings were required.

To enhance methodological rigor, bracketing was applied throughout the study. Prior to data collection and analysis, members of the research team discussed and documented their assumptions and expectations regarding horticultural therapy and post-stroke depression. Reflexive notes were maintained throughout the research process to minimize the potential influence of researchers’ preconceptions on data interpretation.

All interviews were conducted in Mandarin Chinese. Quotations included in this manuscript were translated into English and independently reviewed by two bilingual researchers. Any discrepancies in translation were discussed and resolved through consensus to ensure conceptual accuracy and consistency. Minor linguistic modifications were made to improve clarity while preserving the original meaning and context of participants’ narratives.

### 2.6. Trustworthiness

The rigor of the study was established according to the criteria of credibility, dependability, confirmability, and transferability.

#### 2.6.1. Credibility

Credibility was enhanced through prolonged engagement with participants, use of probing questions during interviews, and member checking following data analysis. Participants were invited to review summaries of the findings to confirm that the interpretations accurately reflected their experiences.

#### 2.6.2. Dependability

An audit trail was maintained throughout the research process, documenting decisions related to participant recruitment, data collection, coding, and theme development. Independent analysis by two researchers further strengthened consistency.

#### 2.6.3. Confirmability

Regular discussions were conducted within the research team to challenge assumptions and minimize researcher bias. Reflexive notes and analytical memos were maintained throughout data analysis.

#### 2.6.4. Transferability

Detailed descriptions of the study setting, participant characteristics, intervention process, and analytical procedures were provided to facilitate assessment of the applicability of findings to other contexts.

### 2.7. Ethical Considerations

Ethical approval was obtained from the Ethics Committee of shibei Hospital, Jing’an District, Shanghai (Approval No. YL-20250619-25), where participant recruitment and data collection were conducted.

All participants received verbal and written information regarding the study objectives, procedures, potential risks, and benefits before enrollment. Participation was entirely voluntary, and participants were informed of their right to withdraw from the study at any time without affecting their treatment.

Confidentiality and privacy were rigorously maintained throughout the study. Personal identifiers were removed from all transcripts, and participant codes (P1–P15) were used in place of names. Audio recordings, transcripts, and analytical documents were stored securely and accessed only by members of the research team.

Participants continued to receive routine medical and rehabilitation care throughout the study period. Although formal suicide risk assessment was not conducted as part of the study protocol, participants were monitored through regular clinical care. If significant psychological distress or concerns regarding participant safety had been identified during interviews or intervention activities, referral to the appropriate clinical team would have been initiated according to hospital procedures.

## 3. Results

A total of 15 patients with PSD completed the study and contributed interview data. Data saturation was achieved after the fifteenth interview, and no new themes emerged during subsequent analysis. Participant characteristics are presented in Table 1. Characteristics of the horticultural therapy program are presented in Table 2. Through Colaizzi’s phenomenological analysis, five themes and ten subthemes were identified, reflecting participants’ experiences of horticultural therapy (Table 3).

### 3.1. Theme 1: Rebuilding Self-Efficacy

Participants consistently reported that horticultural therapy helped restore a sense of competence and control that had been diminished following stroke. Through caring for plants and completing horticultural activities, participants gradually regained confidence in their abilities and developed a stronger sense of self-efficacy.

#### 3.1.1. Transition from Care Recipient to Caregiver

Following stroke, many participants described becoming increasingly dependent on family members and healthcare professionals because of functional limitations. Caring for plants enabled them to assume a nurturing role again and regain a sense of responsibility and purpose.

One participant stated:

“Before my stroke, I never imagined I would become so dependent. At one point, I could barely do anything on my own; even drinking water required assistance. Now I can care for this flower, water it, and clean its leaves. Watching it thrive makes me feel that I am still capable and useful.”

(P3)

Another participant shared:

“I used to be the backbone of my family. After the stroke, I had to rely on my spouse for almost everything, which was difficult to accept. Now, taking care of this plant feels a bit like caring for my children in the past. I feel responsible for its well-being.”

(P7)

For some participants, assuming responsibility for a living plant brought renewed meaning to daily life.

“The plant never judges me for being slow. It simply needs my care. Every time I notice a new leaf growing, I feel that my day has been meaningful.”

(P12)

#### 3.1.2. Restoration of Perceived Competence

Successful horticultural activities helped participants recognize their remaining abilities and regain confidence in performing tasks independently.

“When I first started rehabilitation, I often felt that I would never be able to do things properly again. Horticultural activities were different. Even with one hand, I could still plant a seed. When it finally sprouted, I suddenly realized that I was still capable of accomplishing something.”

(P5)

Another participant described gradual improvement in physical functioning through horticultural activities:

“My right hand is not very flexible, and repotting a plant required both hands. At first, I was clumsy and struggled a lot. Over time, however, I noticed that my hand was becoming more functional. It was not something others told me—I could feel the improvement myself.”

(P9)

### 3.2. Theme 2: Reconstructing Hope for Recovery

Participants frequently associated plant growth with their own rehabilitation journey. Observing plants emerge, grow, and thrive provided a source of hope and motivation during recovery.

#### 3.2.1. Hope Projection Through Plant Growth

Many participants viewed plant growth as a reflection of their own progress and recovery.

“The first time I saw the seedling emerge from the soil, I almost cried. If such a tiny seed can push its way through the earth and grow, then perhaps I can also find my way back from this illness.”

(P1)

Another participant stated:

“My pothos grows a little more every day. Watching it develop reminds me that recovery can also happen gradually. Every morning, the first thing I do is check whether it has grown. It gives me motivation to start the day.”

(P4)

Similarly:

“When I first brought the tomato seedling home, it looked weak and lifeless. I thought it would die. But after regular watering and care, it recovered and eventually flowered. Seeing that gave me strength—I felt that if the plant could recover, maybe I could too.”

(P8)

#### 3.2.2. Perceiving the Resilience of Life

Participants also described drawing strength from the resilience and persistence demonstrated by plants.

“When I first became ill, I sometimes felt that life was no longer worth living. Now, when I see my flowers blooming even through difficult seasons, I am reminded of how resilient life can be. If they can survive, perhaps I can as well.”

(P11)

Another participant reflected:

“When I noticed that a plant could survive after losing several leaves, I began to think differently about my own recovery. Setbacks do not necessarily mean failure. The plant kept growing, and I felt that I should continue trying as well.”

(P6)

Similarly, one participant stated:

“Some plants grew very slowly, but they continued to grow little by little. Watching this process taught me patience and reminded me that my recovery would also take time.”

(P14)

### 3.3. Theme 3: Enhancing Social Connectedness and Sense of Value

Participants reported experiences of increased social interaction and a greater sense of value within interpersonal relationships during their participation in the horticultural therapy program.

#### 3.3.1. Plants as Facilitators of Social Interaction

Plants often became a common topic of conversation and facilitated communication with family members, peers, and healthcare professionals.

“Before I started horticultural activities, my husband and I rarely talked. I often felt guilty because he had to care for me all the time. Since I began growing flowers, he asks me about them every day and we talk much more than before.”

(P2)

Another participant explained:

“We often compare our plants and share tips with each other. When someone’s plant is not doing well, everyone tries to help. It creates a sense of companionship and makes me feel less alone.”

(P6)

#### 3.3.2. Experiencing Value Through Giving

Participants described deriving satisfaction and self-worth from sharing products of their horticultural activities with others.

“When my carnations bloomed, I cut a few flowers and gave them to one of the nurses who cared for me. Seeing her smile made me happy. It reminded me that I can still bring joy to other people.”

(P13)

Similarly:

“I made a bookmark using leaves from the plants I grew and gave it to my grandson. When I heard him proudly tell his classmates that it was made by his grandmother, I felt deeply touched.”

(P10)

### 3.4. Theme 4: Perceived Contributions to Physical and Psychological Rehabilitation

Participants perceived horticultural therapy as a meaningful activity that simultaneously supported physical rehabilitation and emotional well-being.

#### 3.4.1. Increased Engagement in Rehabilitation Activities

Many participants reported being more willing to engage in physical activity during horticultural activities than during conventional rehabilitation exercises.

“Traditional rehabilitation can be exhausting, and sometimes I do not feel motivated to do it. Horticultural activities is different. Because I want the plant to grow well, I naturally use my affected hand to hold the pot or pick up tools. Afterwards, I realize that I have actually been exercising without noticing it.”

(P3)

Another participant stated:

“Watering the plants, bending down, and reaching up to prune them all require movement. While I was doing these activities, I was not thinking about rehabilitation at all. Later I noticed that my body felt less stiff than before.”

(P14)

#### 3.4.2. Emotional Regulation Through Multisensory Stimulation

Participants described positive emotional responses associated with visual, olfactory, and tactile experiences during horticultural activities.

“The smell of soil and flowers makes me feel calm and peaceful. I used to lie awake at night worrying about many things, but now these scents help me feel more settled and relaxed.”

(P7)

Another participant reported:

“I like touching the soft, fuzzy leaves. They feel comforting. Whenever I am feeling upset, simply touching them seems to ease some of the emotional burden.”

(P15)

### 3.5. Theme 5: Challenges and Negative Experiences During Horticultural Activities

Although participants generally described horticultural therapy positively, they also reported challenges and negative experiences associated with horticultural activities.

#### 3.5.1. Frustration Associated with Unsuccessful Plant Growth

Failure of plants to thrive occasionally led to disappointment, frustration, and self-doubt.

“My mint plant never grew well and eventually died. I felt as though I had failed to take care of it properly. It was discouraging because it reminded me of my own recovery, where progress sometimes feels very slow despite my efforts.”

(P5)

Similarly:

“When I saw other people’s flowers flourishing while mine showed little growth, I began to question myself. I wondered whether I was somehow less capable than others. If I could not even grow a plant successfully, what else could I do?”

(P1)

#### 3.5.2. Re-Emergence of Helplessness Due to Physical Limitations

Persistent physical impairments sometimes prevented participants from independently completing gardening and contributed to feelings of helplessness.

“I wanted to repot the plant by myself, but my hand was too weak. No matter how hard I tried, I could not manage it alone and eventually needed help from a nurse. At that moment, I felt helpless and disappointed in myself.”

(P9)

Another participant reported:

“I saw other patients making beautiful handicrafts and wanted to do the same. However, my hands trembled too much and the things I made did not turn out well. After that, I felt discouraged and lost motivation to continue.”

(P12)

Overall, participants described horticultural therapy as a multifaceted experience that contributed to psychological empowerment, social engagement, and rehabilitation participation while simultaneously presenting challenges that required ongoing support and adaptation.

## 4. Discussion

This study explored the lived experiences of patients with post-stroke depression who participated in a horticultural therapy program. Empowerment Theory provided a useful framework for interpreting the findings. Participants described experiences of increased confidence, renewed hope, enhanced social connectedness, and greater engagement in rehabilitation activities in the context of their participation in the horticultural therapy program. At the same time, participants also described challenges associated with unsuccessful plant growth and persistent physical limitations, highlighting the need for individualized support throughout the intervention process.

### 4.1. Horticultural Therapy as a Pathway to Psychological Empowerment

A key finding of this study was that participants perceived a gradual restoration of self-efficacy in the context of caring for living plants. Following stroke, many participants described feelings of dependence, reduced competence, and loss of valued social roles. Through activities such as sowing seeds, watering plants, and monitoring plant growth, participants were able to experience mastery, responsibility, and achievement. Participants described these experiences as contributing to a sense of increased involvement in their rehabilitation process.

From an empowerment perspective, these findings reflect participants’ perceptions of enhanced confidence, perceived competence, and personal control. Participants frequently interpreted successful plant cultivation as evidence that they remained capable despite their disabilities. Such mastery experiences are particularly meaningful for individuals with PSD [1,4], whose depressive symptoms are often accompanied by helplessness, uncertainty, and diminished self-worth. Participants perceived horticultural activities as meaningful experiences that were associated with renewed confidence and engagement in rehabilitation-related activities [20,21,22].

### 4.2. Cultivating Hope Through Symbolic Experiences of Growth

Another prominent finding was the symbolic meaning participants attached to plant growth. Some participants viewed the processes of germination, development, and flowering as symbolic parallels to their own recovery journeys. Participants described drawing encouragement from observing gradual changes in plants and interpreting these changes as evidence that recovery is possible despite setbacks.

Hope is considered an essential component of psychological recovery and successful adaptation to chronic illness. For many participants, horticultural activities provided tangible and visible representations of progress that contrasted with the often slow and uncertain nature of stroke rehabilitation. The growth of plants appeared to provide a tangible representation of personal progress and adaptation, thereby fostering optimism and future-oriented thinking. These findings illustrate how participants interpreted horticultural activities as meaningful experiences that supported their sense of hope, without establishing therapeutic mechanisms or causal effects [23,24,25].

### 4.3. Strengthening Social Connectedness and Personal Value

Participants also described horticultural therapy as a valuable social experience. Horticultural activities created opportunities for interaction with family members, peers, and healthcare professionals. Several participants described discussions about plant growth, sharing cultivation experiences, and exchanging horticultural activities as meaningful opportunities for social interaction and a renewed sense of belonging.

An interesting finding was that participants experienced a strong sense of value through giving flowers, handicrafts, and other horticultural activities to others. Following stroke, many individuals experience role loss and perceive themselves primarily as recipients of support. The opportunity to contribute to the well-being of others appeared to restore a sense of usefulness and social identity. These findings illustrate how participants interpreted horticultural activities as meaningful experiences that supported their sense of value and social identity, without establishing therapeutic mechanisms or causal effects [26,27,28].

### 4.4. Implications for Mental Health Recovery and Rehabilitation

Participants consistently described horticultural therapy as more meaningful and enjoyable than traditional rehabilitation exercises. Unlike conventional therapy, horticultural activities integrated physical movement, emotional engagement, sensory stimulation, and purposeful activity into a single experience. Participants often reported that they engaged more willingly in physical activity when it was connected to caring for plants rather than completing prescribed exercises [29,30].

Participants’ narratives reflected their perceptions of how horticultural activities related to psychosocial aspects of their recovery, rather than indicating therapeutic mechanisms. Interaction with nature appeared to promote relaxation and emotional regulation, while horticultural activities provided opportunities for achievement and social participation. These multidimensional benefits align with contemporary recovery-oriented approaches in mental health nursing, which emphasize strengths, meaningful engagement, hope, and participation rather than symptom reduction alone. Participants perceived horticultural therapy as a valuable complement to their rehabilitation experiences and psychological adjustment following stroke [31,32]. It should be noted that symbolic interpretations of plant growth, such as viewing it as a source of hope or a metaphor for recovery, were described by some participants but were not consistent across the entire sample.

### 4.5. Negative Experiences and the Need for Individualized Nursing Support

Although participants overwhelmingly described positive experiences, the findings also revealed important challenges. Plant death, unsuccessful cultivation outcomes, and physical difficulties occasionally triggered disappointment, self-doubt, and renewed feelings of helplessness. For some participants, horticultural activity setbacks were interpreted as indicators of personal failure and were closely linked to concerns about their own recovery.

These findings highlight that horticultural therapy should not be viewed as universally beneficial without appropriate support. Mental health and rehabilitation nurses play an important role in helping participants interpret setbacks constructively, normalize difficulties encountered during plant cultivation, and maintain realistic expectations. Individualized activity selection, graded task difficulty, ongoing encouragement, and emotional support may help participants maintain positive engagement while minimizing potential distress.

### 4.6. Implications for Psychiatric and Mental Health Nursing

The present findings have several implications for psychiatric and mental health nursing practice. First, participants perceived horticultural activities as meaningful experiences that contributed to feelings of hope, self-efficacy, and social engagement. Second, participants’ experiences suggest that horticultural activities may offer opportunities for meaningful participation and empowerment within recovery-oriented care. Third, participants described group-based horticultural activities as providing opportunities for social interaction and peer support. Finally, nurses should recognize potential emotional reactions to unsuccessful horticultural activities and provide timely psychological support when needed.

It is important to acknowledge that this phenomenological study was designed to explore participants’ lived experiences rather than evaluate intervention efficacy. Participants’ descriptions of perceived changes may also have been influenced by factors beyond horticultural therapy, including natural recovery following stroke, ongoing rehabilitation services, social support, and concurrent pharmacological or psychological treatments. Therefore, the findings should be interpreted as subjective experiences rather than evidence of causal effects.

Participants also received multidisciplinary rehabilitation services throughout the study period, including rehabilitation therapy, nursing care, psychological support, peer interaction, and group-based activities. In particular, some positive experiences may have been attributable to group interaction, attention from healthcare professionals, physical activity, outdoor or nature exposure, or general rehabilitation engagement, rather than the horticultural activities themselves. Information on concurrent antidepressant medication or psychotherapy was not systematically collected as part of this study, although available medical records indicated that no major changes in depression-related treatment occurred during the horticultural therapy period.

Only participants who completed the six-month horticultural therapy program were eligible for inclusion in this study. Individuals who discontinued participation before program completion were not represented and may have experienced different barriers, levels of engagement, or perceptions of benefit. Consequently, survivor bias cannot be excluded, and the findings may disproportionately reflect the experiences of participants who were willing and able to remain engaged in the intervention throughout the study period.

Furthermore, participants with severe aphasia, major communication impairment, severe cognitive dysfunction, ADL ≤ 40, or muscle strength ≤ Grade III were excluded from participation. Therefore, the findings primarily reflect the experiences of relatively communicative and functionally preserved stroke survivors. Individuals with more severe post-stroke disability may experience horticultural therapy differently, and caution should be exercised when transferring these findings to more severely affected populations. This exclusion criterion may have systematically omitted individuals who could experience horticultural therapy differently, thereby limiting the diversity of perspectives represented in this study.

In addition, all interviews were conducted within the hospital where the horticultural therapy programme was delivered, which may have contributed to social desirability bias or influenced participants’ willingness to disclose negative or contradictory experiences. Although both positive and challenging experiences were explored in the interviews, participants tended to emphasize perceived benefits more frequently than difficulties, which may have influenced the balance of themes presented in the manuscript.

Taken together, the single-centre design, small purposively selected sample, absence of a comparison group, and potential selection and response biases substantially limit the transferability of the findings. These methodological constraints should be considered when interpreting the results and when applying them to other stroke rehabilitation settings or populations.

## 5. Conclusions

Horticultural therapy was experienced by participants with post-stroke depression as a meaningful and empowering experience within their rehabilitation journey. Through caring for plants, participants reported experiencing greater self-efficacy, renewed hope, enhanced social connectedness, and increased engagement in rehabilitation activities. At the same time, negative experiences associated with plant loss and physical limitations highlighted the need for individualized support during horticultural activities.

The findings reflect participants’ subjective experiences and perceptions of horticultural therapy rather than evidence of clinical efficacy. Participants described horticultural therapy as contributing to feelings of empowerment, hope, and social participation within the context of their broader rehabilitation. Based on these experiential accounts, horticultural activities may hold potential implications for recovery-oriented mental health and rehabilitation nursing, particularly in creating opportunities for meaningful engagement and supporting psychosocial well-being. However, these insights should be interpreted as exploratory and not as recommendations for routine clinical implementation.

## 6. Relevance for Clinical Practice

Horticultural therapy was described by participants as a feasible and person-centered activity within rehabilitation settings that offered perceived psychosocial benefits. Participants’ accounts suggested that horticultural activities may support feelings of self-efficacy, hope, social connectedness, and active engagement in recovery. These experiential insights indicate that horticultural activities could hold potential value for rehabilitation and mental health nursing, particularly in creating opportunities for meaningful participation and fostering recovery-oriented interactions.

Participants also emphasized the importance of individualized support, noting that plant loss, unsuccessful cultivation, and physical limitations could lead to frustration or self-doubt. From a nursing perspective, these experiences highlight the potential relevance of providing emotional support and adapting activities to participants’ functional abilities when horticultural activities are used within rehabilitation contexts.

Overall, these findings offer exploratory implications for how nature-based activities might inform holistic, recovery-oriented psychosocial care for individuals with post-stroke depression. However, they should not be interpreted as evidence of treatment efficacy or as recommendations for routine clinical implementation.

## Figures and Tables

**Table 1:** Sociodemographic and Clinical Characteristics of Participants (*N* = 15).

Characteristic	Value
Total participants	15
Age (years), mean ± SD	62.3 ± 8.7
Male, *n* (%)	9 (60.0)
Female, *n* (%)	6 (40.0)
Ischemic stroke, *n* (%)	11 (73.3)
Hemorrhagic stroke, *n* (%)	4 (26.7)
Left hemiplegia, *n* (%)	8 (53.3)
Right hemiplegia, *n* (%)	7 (46.7)
Depression severity	Mild-to-moderate PSD (HAMD-17 score 8–24)
Participation in horticultural therapy	≥6 months
Functional status	ADL > 40; Muscle strength > Grade III

**Table 2:** Characteristics of the Horticultural Therapy Program.

Component	Description
Duration	6 months
Frequency	Weekly
Session length	60–90 min
Format	Individual and small-group activities
Facilitators	Nurses, rehabilitation therapists, horticultural instructors, counselors
Main activities	Planting, watering, pruning, transplanting, flower arrangement, handicrafts
Therapeutic goals	Enhance empowerment, hope, social participation, and rehabilitation engagement

**Table 3:** Themes and Subthemes Identified From Participants’ Experiences of Horticultural Therapy.

Themes	Subthemes
Theme 1. Rebuilding Self-Efficacy	Transition from Care Recipient to Caregiver
	Restoration of Perceived Competence
Theme 2. Reconstructing Hope for Recovery	Hope Projection Through Plant Growth
	Perceiving the Resilience of Life
Theme 3. Enhancing Social Connectedness and Sense of Value	Plants as Facilitators of Social Interaction
	Experiencing Value Through Giving
Theme 4. Facilitating Physical and Psychological Rehabilitation	Increased Engagement in Rehabilitation Activities
	Emotional Regulation Through Multisensory Stimulation
Theme 5. Challenges and Negative Experiences During Horticultural Engagement	Frustration Associated With Unsuccessful Plant Growth
	Re-emergence of Helplessness Due to Physical Limitations

## Data Availability

The data presented in this study are available on request from the corresponding author due to privacy and ethical restrictions related to patient data.

## Supplementary Materials

The following supporting information can be downloaded at: https://www.mdpi.com/article/10.3390/healthcare14193229/s1.
