# Supplementary material for: Reconstructing Hope Through Horticultural Therapy: A Phenomenological Study of Individuals with Post-Stroke Depression
Source: Healthcare (Basel). 2026 Oct 1;14(19):3229. doi: 10.3390/healthcare14193229 (PMC13664736; doi:10.3390/healthcare14193229)
Supplement: Supplementary file 1 [file healthcare-14-03229-s001.zip › healthcare-4524661-supplementary.pdf]

Supplementary Material S1

Semi-Structured Interview Guide

Before the interview

Participants were informed about the purpose of the study and encouraged to freely share their experiences of participating in the horticultural therapy programme. Follow-up questions were used when appropriate to facilitate deeper exploration of participants' experiences.

Opening Question

1. Please describe your overall experience of participating in the horticultural therapy programme.

Main Interview Questions

1. How have your emotions and psychological state changed since participating in horticultural activities?
2. Can you describe any memorable experiences during the process of planting or caring for plants?
3. In what ways, if any, do you think horticultural therapy influenced your rehabilitation experience?

1. Have you encountered any difficulties, frustrations, or challenges during the horticultural activities? How did you deal with them?
2. How would you describe the relationship between yourself and the plants you cared for?
3. What aspects of the horticultural activities were most meaningful to you?

Probing Questions

The following probing questions were used when appropriate:

- Could you tell me more about that experience?
- How did that make you feel?
- Why was that important to you?
- Can you give an example?
- What were your thoughts at that time?
- Has your perspective changed since then?

Closing Question

1. Is there anything else you would like to share about your experience of participating in the horticultural therapy programme?

Supplementary Material S2

Example of the Coding and Theme Development Process

Theme 1: Reconstructing a Positive Sense of Self

Subtheme 1.1: Regaining a Sense of Accomplishment

Participant quotation

"When I saw the seeds I planted gradually sprout and bloom, I felt that I had accomplished something. It made me feel more confident."

Significant statement

The participant experienced confidence and achievement through observing plant growth.

Formulated meaning

Successful plant cultivation fostered positive self-evaluation.

Code

Sense of accomplishment

Subtheme

Regaining a Sense of Accomplishment

Theme

Reconstructing a Positive Sense of Self

Theme 2: Perceiving the Resilience of Life

Subtheme 2.1: Learning from Plant Growth

Participant quotation

"Even after being damaged, the plants continued to grow. I felt that I should not give up either."

Significant statement

The participant related plant resilience to personal recovery after stroke.

Formulated meaning

Plants served as symbols of persistence and recovery.

Code

Learning resilience from nature

Subtheme

Learning from Plant Growth

Theme

Perceiving the Resilience of Life

Theme 3: Reconnecting with Others and the Environment

Subtheme 3.1: Developing Social Connections

Participant quotation

“We often discussed how to take care of the plants. Talking with other patients made me feel less isolated.”

Significant statement

The participant gained social interaction through horticultural activities.

Formulated meaning

Shared horticultural experiences enhanced social engagement.

Code

Improved social interaction

Subtheme

Developing Social Connections

Theme

Reconnecting with Others and the Environment
